# Role of Endoplasmic Reticulum Aminopeptidases in Health and Disease: from Infection to Cancer

**DOI:** 10.3390/ijms13078338

**Published:** 2012-07-04

**Authors:** Loredana Cifaldi, Paolo Romania, Silvia Lorenzi, Franco Locatelli, Doriana Fruci

**Affiliations:** 1 Paediatric Haematology/Oncology Department, Bambino Gesù Children’s Hospital IRCCS, Piazza S. Onofrio 4, Rome 00165, Italy; E-Mails: loredana.cifaldi@opbg.net (L.C.); paolo.romania@opbg.net (P.R.); silvia.lorenzi@opbg.net (S.L.); franco.locatelli@opbg.net (F.L.); 2 University of Pavia, Corso Strada Nuova 65, Pavia I-27100, Italy

**Keywords:** endoplasmic reticulum aminopeptidases, MHC class I, antigen processing, autoimmunity, viral infection, cancer, cancer immunotherapy

## Abstract

Endoplasmic reticulum (ER) aminopeptidases ERAP1 and ERAP2 (ERAPs) are essential for the maturation of a wide spectrum of proteins involved in various biological processes. In the ER, these enzymes work in concert to trim peptides for presentation on MHC class I molecules. Loss of ERAPs function substantially alters the repertoire of peptides presented by MHC class I molecules, critically affecting recognition of both NK and CD8^+^ T cells. In addition, these enzymes are involved in the modulation of inflammatory responses by promoting the shedding of several cytokine receptors, and in the regulation of both blood pressure and angiogenesis. Recent genome-wide association studies have identified common variants of ERAP1 and ERAP2 linked to several human diseases, ranging from viral infections to autoimmunity and cancer. More recently, inhibition of ER peptide trimming has been shown to play a key role in stimulating innate and adaptive anti-tumor immune responses, suggesting that inhibition of ERAPs might be exploited for the establishment of innovative therapeutic approaches against cancer. This review summarizes data currently available for ERAP enzymes in ER peptide trimming and in other immunological and non-immunological functions, paying attention to the emerging role played by these enzymes in human diseases.

## 1. Introduction

The endoplasmic reticulum (ER) aminopeptidase 1 (ERAP1) and the closely related ER aminopeptidase ERAP2 are zinc-metallopeptidases of the oxytocinase M1 subfamily, which share consensus zinc-binding motifs essential for their enzymatic activity [1]. The human ERAP1 and ERAP2 genes are located on chromosome 5q15 in the opposite orientation, likely to share regulatory elements. Human ERAP2 has no analogues in rodents (e.g., mouse, rat, rabbit and guinea pig) and evolution studies suggest that it originates from a relatively recent duplication of ERAP1 [2]. These enzymes are normally present in many tissues and are strongly induced after stimulation with type I and type II interferons (IFNs) [3–6] and tumor necrosis factor-alpha (TNF-α) [7].

**Figure 1:**
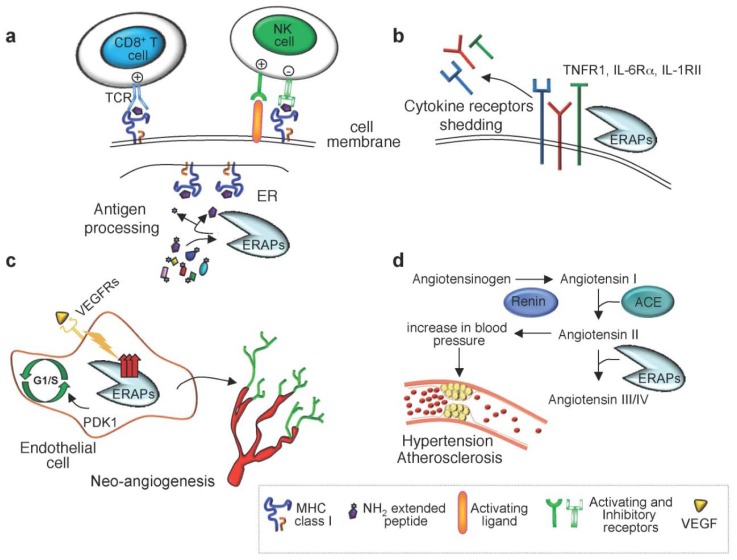
Schematic representation of the multifunctional properties of endoplasmic reticulum (ER) aminopeptidase 1 (ERAP1) and ERAP2. ERAP1 and ERAP2 (ERAPs) are involved in a variety of biological processes including (**a**) the final trimming of peptides in the endoplasmic reticulum (ER) for presentation on MHC class I molecules; (**b**) shedding of several cytokine receptors; (**c**) post-natal angiogenesis; (**d**) regulation of blood pressure. For details see text.

ERAP enzymes trim amino acid residues from the NH_2_ terminus of polypeptides playing an important role in various biological processes (Figure 1). In the ER, ERAP1 and ERAP2 cleave precursors to generate or destroy MHC class I binding peptides [5,8,9]. ERAP1 has also been involved in regulation of innate immune and inflammatory responses by increasing the shedding of cytokine receptors [10–12]. In addition to these immunological functions, ERAP1 and ERAP2 have been implicated in the regulation of angiogenesis and blood pressure [6,13,14]. According to these multifunctional properties, ERAP1 is also termed endoplasmic reticulum aminopeptidase associated with antigen processing (ERAAP), adipocyte-derived leucine aminopeptidase (A-LAP), puromycin-insensitive leucine-specific aminopeptidase (PILS-AP), or aminopeptidase regulating type I TNF receptor (TNFR1) shedding (ARTS-1), whereas ERAP2 is known as leukocyte-derived arginine aminopeptidase (L-RAP). The terms ERAP1 and ERAP2, approved by the Human Genome Organization Nomenclature Committee, will be used in this review.

## 2. Immunological Functions of ER Aminopeptidases

MHC class I binding peptides are generated by the antigen-processing pathway through a series of sequential steps [15]. In the first step, endogenous proteins are degraded in the cytosol through the proteasome, a multicatalytic complex that generates fragments with hydrophobic or positively charged COOH-terminal anchor residues. Most of these fragments are further processed by cytosolic aminopeptidases, tripeptidyl peptidase II [16–18], leucine aminopeptidase [19,20], bleomycin hydrolase and puromycin-sensitive aminopeptidase [21,22], or directly translocated into the ER lumen by the transporter associated with antigen processing (TAP), an ATP-driven transporter well adapted for the transfer of these precursor peptides [23]. In the ER, the NH_2_ terminus of these peptides is further trimmed by ER aminopeptidases, *i.e.*, ERAP1 in mice and ERAP1 and ERAP2 in humans, to the proper length for binding to MHC class I molecules and presentation on the cell surface for recognition by NK cells and specific CD8^+^ T cells (Figure 1a) [5,8,9].

### 2.1. Trimming of Antigenic Peptides by ER Aminopeptidases

ERAP1 efficiently trims peptides of 9–16 amino acids, the length of peptides efficiently transported into the ER by TAP, but spares the longer ones [24]. Of note, its activity is substantially reduced for peptides with proline at position 2 (X-P-X_n_) [5,25], or for peptides with a size of 8 or 9 amino acids, the optimal length for binding to MHC class I molecules [24]. ERAP1 shows a strong preference for peptides with large hydrophobic COOH-terminal residues [24]. ERAP1 activity appears to be also affected by the nature of the internal residues of peptides. In particular, positions 2, 5 and 7 (with position 1 defined as the N-terminal residue of the peptide) were found to be the most important for the peptide sensitivity to ERAP1 degradation [26]. Based on the analogies with TAP and MHC class I preferences, Chang *et al.* proposed the “molecular ruler” model for ERAP1 [24], that suggests its role in facilitating antigen processing and presentation by trimming precursors transported by TAP to MHC class I binding peptides.

ERAP2 has been shown to cooperate with ERAP1 to trim a large variety of precursor peptides to generate mature epitopes for binding to MHC class I molecules [9]. ERAP2 was found to have distinct specificities for the N-terminal residue of the peptide substrates and to physically associate with ERAP1. This complex is expected to be more efficient than single enzymes in dealing the large number of precursor peptides. To date, there are few studies regarding ERAP2 function.

The substantial contribution of ER peptide trimming to MHC class I antigen processing and presentation has been confirmed in mice lacking ERAP1 generated independently in four laboratories [27–30]. Although loss of ERAP1 had a relatively modest effect on the cell surface expression of most MHC class I molecules (a reduction of 20–40% for K^b^ and D^b^ class I molecules) [31], immunization of ERAP1^−/−^ mice with wild-type (wt) cells or *vice versa*, resulted in potent CD8^+^ T cell responses, suggesting that loss of ERAP1 alters the peptide-MHC (pMHC) class I repertoire not only quantitatively but also qualitatively [27]. Analysis of the individual peptides displayed on the cell surface with a panel of peptide-specific CD8^+^ T cell hybridomas showed that ERAP1 deficiency left some peptides unaffected, whereas others were either absent or dramatically up-regulated [5,27–32]. Consistent with these findings, mass spectrometry analysis of natural and viral peptides processed in mice lacking ERAP1, revealed that ERAP1 deficiency causes a marked increase in the length of peptides normally presented by MHC class I molecules [33]. Thus, ERAP1 proteolysis determines the characteristic length, as well as the composition of MHC class I binding peptide in the ER.

In addition to classical MHC class I molecules, ERAP1^−/−^ mice also exhibited defects in the surface expression of nonclassical MHC class I molecules Qa-2 and Qa-1^b^, which serve as ligands in both the innate and adaptive immune responses [28]. Yan and collaborators found a significant reduction of the nonclassical class I molecules Qa-2 in ERAP1-deficient splenocytes and dendritic cells as compared with wt cells. Although ERAP1 did not significantly affect the surface expression of Qa-1 molecules, presentation of Qdm, an epitope derived from the signal sequence of classical MHC class I molecules, to Qa-1-restricted CTLs was impaired, suggesting that ERAP1 activity is required for the generation of this epitope. Thus, it is conceivable that reduced ERAP1 function may represent a rate-limiting step in presenting Qdm peptide to Qa-1^b^-restricted CD8^+^ T cells or NK cells expressing CD94/NKG2 receptors.

More recently, a new naturally processed Qa-1^b^ epitope (FL9) derived from the Fam49b protein, has been identified in cells lacking ERAP1 activity by Shastri and colleagues [34]. Unlike the Qa-1^b^-Qdm complex, Qa-1^b^-FL9 is an immunodominant ligand recognized by CD8^+^ T cells derived from wt mice immunized with ERAP1 deficient cells. The authors found an abundant fraction of CD8^+^ T cells specific for the Qa-1^b^-FL9 complex in naive wt mice able to proliferate and efficiently eliminate ERAP1-deficient cells.

### 2.2. Cytokine Receptor Shedding

In addition to antigen processing, ERAP1 has been shown to promote the cleavage of several cytokine cell surface receptors (Figure 1b) [10–12]. Cui *et al.* demonstrated that ERAP1 binds to the extracellular domain of the TNFR1, facilitating TNFR1 shedding through the formation of a TNFR1/ERAP1 complex. The authors showed that overexpression of ERAP1 produces soluble TNFR1 that competes with cell-surface TNF receptors, thereby attenuating TNFα bioactivity when the levels are elevated, and reconstituting TNFα when the levels have declined [10]. However, several evidences demonstrated that ERAP1 is not required to be catalytically active for this function. Coimmunoprecipitation experiments revealed that ERAP1 can bind to, but not cleave TNFR1. Moreover, ERAP1 does not possess endopeptidase activity and overexpression of ERAP1 catalytic mutants results in an increased TNFR1 shedding. Thus, ERAP1 does not directly catalyze TNFR1 shedding, but may instead promote the activity of a TNFR1 sheddase.

Subsequently, the same authors showed that ERAP1 modulates the proteolytic cleavage of two other cytokine receptors, the type I IL-6 cytokine receptor (IL-6Rα) [12] and the type II IL-1 decoy receptor (IL-1RII) [11]. Based on these functions, ERAP1 has been proposed to play an important role in regulating innate and inflammatory immune responses by increasing the shedding of these cytokine receptors.

## 3. Nonimmunological Functions of ER Aminopeptidases

ERAP1 and ERAP2 are thought to play a role in the regulation of blood pressure through their involvement in the renin-angiotensin system (Figure 1d). *In vitro* studies using Chinese Hamster Ovary cells demonstrated that ERAP1 efficiently cleaves angiotensin II to angiotensin III and IV [14], while ERAP2 cleaves angiotensin III to angiotensin IV [6]. In the same system, both enzymes were shown to convert kallidin to bradykinin [6,14].

In addition, ERAP1 has been reported to control post-natal neo-angiogenesis, a physiological process involving the growth of new blood vessels from pre-existing microvessels, by regulating the proliferation and migration of endothelial cells (EC) (Figure 1c) [35–39]. Functional studies revealed that ERAP1 is expressed in ECs during differentiation *in vitro* and at the site of angiogenesis *in vivo* upon stimulation with vascular endothelial growth factor (VEGF) [35]. Suppression of ERAP1 expression in ECs inhibited VEGF-stimulated proliferation, migration and vessel network formation *in vitro* and angiogenesis *in vivo* [35]. The authors also demonstrated that ERAP1 regulates VEGF-stimulated G1/S transition during EC proliferation by binding to phosphatidylinositol-dependent kinase 1 (PDK1). Formation of the ERAP1-PDK1-S6 kinase complex resulted in activation of cyclin-dependent kinase (CDK) 4/6 by phosphorylated S6K that promotes G1/S-phase transition leading to EC proliferation [37]. It was also demonstrated that ERAP1 controls the spreading of ECs by activating endothelial integrins and focal adhesion kinase [36], increasing EC adhesion to the extracellular matrix via RhoA activation [38]. More recently, ERAP1 has also been shown to bind pigpen, a nuclear coiled body component protein involved in angiogenesis [39,40]. However, how pigpen interacts with ERAP1 to promote angiogenesis and whether pigpen is a substrate for ERAP1 remain to be established.

## 4. Alteration of ERAP Functions in Human Diseases

ERAP1 and ERAP2 are highly polymorphic genes. Naturally occurring single nucleotide polymorphisms (SNPs) have been found to correlate with several pathological conditions [41–43]. Because of the role of these enzymes in MHC class I antigen presentation and in all other biological processes, it is conceivable that variations impairing the presentation of pathogen-derived peptides might lead to inadequate immune responses and development of disease. Recent studies that investigated the possible role of ER aminopeptidases in human diseases are summarized below.

### 4.1. Hypertension

By screening for 33 polymorphisms in the human ERAP1 gene, Yamamoto *et al.* identified the association of ERAP1 variant rs30187 (K528R) with essential hypertension and hypothesized that the R528 form of ERAP1 was less active than the K528 form, leading to hypertension due to reduced bradykinin formation and/or lower inactivation of angiotensin II (Figure 1d) [43]. This variant was shown to reduce by 60% the efficiency of ERAP1 to cleave angiotensin II to angiotensin III and by 70% to convert kallidin into bradykinin [44]. The ERAP1 rs30187 variant was also found to determine the degree of regression of left ventricular hypertrophy during anti-hypertensive treatment in patients with essential hypertension [45]. The same variant was also associated with haemolytic uremic syndrome (HUS), a disorder characterized by thrombotic microangiopathy frequently caused by gastrointestinal infections with *Escherichia coli* that produce verotoxins or Shiga toxins [42].

More recently, variants of ERAP1 and ERAP2 have been found to be associated with an increased risk of preeclampsia, a heritable pregnancy specific disorder characterized by new-onset hypertension and proteinuria [46,47]. Of note, ERAP2 expression was previously found altered in first trimester placentas of women prone to develop preeclampsia [48].

### 4.2. Bacterial and Viral Infections

The first evidence demonstrating that ERAP1 can act as a “susceptibility factor” for an infectious organism derived from the observation that ERAP1-deficient mice are not able to process the immunodominant decapeptide HF10 of *Toxoplasma gondii* and die from overwhelming infection when challenged with this pathogen [49].

ERAP1 plays an important role in immune response to viruses, either enhancing or reducing CD8^+^ T-cell responses to particular viral epitopes. ERAP1-deficient or wt mice infected with lymphocytic choriomeningitis virus (LCMV) showed profound differences in the frequency of CD8^+^ T cells specific for particular LCMV peptides [30]. In wt mice the magnitude of T-cell responses to different LCMV epitopes followed a hierarchy of immunodominance that is markedly changed in the ERAP1-deficient mice [30].

Draenert *et al.* reported the first evidence that escape mutations arising in flanking regions of a human immunodeficiency virus (HIV) epitope alter antigen processing mediated by ERAP1 [50]. The authors showed that in HLA-B57^+^-HIV infected individuals, immune selection pressure leads to a mutation from alanine to proline at residue 146 of HIV Gag protein immediately before the NH_2_ terminus of a dominant HLA-B57-restricted CTL epitope. This mutation was found to prevent the NH_2_-terminal cleavage by ERAP1, resulting in decreased CTL responses [50]. Of note, it was demonstrated that antigen processing shapes CTL response hierarchies, and that HIV evolution modifies cleavage patterns influencing proteasomal cleavage and, hence, the likelihood of CTL responses toward all epitopes [51]. Interestingly, some variants in ERAP2 have been shown to confer resistance to HIV-1 infection possibly via the presentation of a distinctive peptide repertoire to CD8^+^ T cells [52].

Similarly, in cervical carcinoma induced by persistent infection and malignant transformation of the uterine cervical epithelium by human papillomavirus (HPV), increased cancer metastasis and decreased survival have been reported to be associated with several variants of ERAP1. In this case, down-regulation of ERAP1 may lead to the preferential loading and presentation of non tumor-associated or non HPV-associated peptides, thereby yielding a less immunogenic phenotype and facilitating tumor growth and progression [53].

Recently, ERAP1 has been identified as a host target of human cytomegalovirus (HCMV) microRNA miR-US4–1, demonstrating a previously unknown miRNA-based immunoevasion strategy [54]. Viral miR-US4–1 interferes with MHC class I-mediated antigen presentation by targeting ERAP1, thereby influencing the production of many HCMV-derived antigenic peptides during viral infection, which results in immunoevasion of the recognition of viral antigen by CD8^+^ T cells during the host immune response [54].

### 4.3. Autoimmune Diseases

Recent genome-wide association studies (GWAS) have proven the importance of ERAP1 and ERAP2 genes in conferring susceptibility of individuals to different autoimmune diseases and their linkage with particular MHC class I alleles [41].

The first GWAS realized by the Wellcome Trust Case Control Consortium and the Austro-Anglo-American Spondylitis Consortium has revealed that 26% of the overall risk to develop ankylosing spondylitis (AS) is accounted for by ERAP1 [55]. The association between AS and ERAP1 variants has been subsequently replicated in other GWAS and several case-control independent studies in Caucasian and Mongolian populations [56–59]. More recently the association with the variant rs30187 has been exclusively found in the cohort of HLA-B27-positive AS patients [60,61]. Of note, the disease-associated ERAP1 variant rs30187 (K528) had faster rate of trimming of peptide precursors than protective ERAP1 variant [60,62]. Taken together, these findings support a model in which aberrant peptide trimming by ERAP1 and, as consequence, impaired peptide presentation by HLA-B27 are involved in the AS pathogenesis [60].

Of note, the functional ERAP1 variant (rs30187) linked to AS, hypertension and HUS was associated with type 1 diabetes and multiple sclerosis [63,64]. No evidence for an interaction between ERAP1 and MHC class I was observed in diabetic patients [63]. Two other recent GWAS identified ERAP1 as a new psoriasis susceptibility locus and evidence of an interaction between HLA-C*06:02 and *ERAP1* was reported [65,66]. Both studied revealed that ERAP1 influences psoriasis susceptibility only in individuals carrying the *HLA-C* risk allele. A recent meta-analysis of six Crohn’s disease GWAS identified ERAP2 as one of the most interesting candidate genes [67].

### 4.4. Cancer

The expression and tissue distribution of ERAP1 and ERAP2 have been evaluated in a large number of tumor cells of lymphoid and non-lymphoid origin compared to their normal counterparts [68–71]. We found that ERAP1 and ERAP2 were expressed in essentially all tumor cell lines examined (melanomas, leukemia-lymphomas and carcinomas of breast, colon, lung, chorion, skin, prostate, cervix, kidney and bladder) at highly variable levels and independently of each other [68]. MHC class I surface expression was significantly correlated with ERAP1, but not with ERAP2, suggesting that ERAP1 has a dominant role in the generation of MHC class I epitopes [68]. In a subsequent study, ERAP1 and ERAP2 were investigated in a large panel of surgically removed normal and neoplastic tissues. In approximately 150 neoplastic lesions, the expression of either or both enzymes was lost, acquired or retained as compared to the normal counterparts, depending on the tumor histotype [69]. Down-regulation of ERAP1 and/or ERAP2 expression was mainly detected in ovary, breast and lung carcinomas, whereas an up-regulation of these enzymes was observed in colon and thyroid carcinomas [69]. Of note, ERAP1 and MHC class I were co-ordinately expressed in normal and, to a lesser extent, neoplastic lesions. As expected, the altered expression of ERAPs results in abnormal cell surface expression of MHC class I molecules in tumor cell lines [68]. In the most aggressive type of neuroblastoma cells, ERAP1, ERAP2 as well as MHC class I molecules were expressed at very low levels as consequence of a poor constitutive NF-kB nuclear activity [7].

In a recent study, heterogeneous expression of ERAP1 and ERAP2, ranging from high to very low levels, was detected in 28 melanoma cell lines as compared to primary melanocytes [70]. In most cases, expression of these genes was enhanced by IFN-γ treatment, suggesting that it is under control of regulatory mechanisms and that only in rare cases to abnormalities in their sequences [70].

Expression of ERAP1 has been detected in 64% of endometrial carcinomas and correlated with CA-125 levels, suggesting a role of this enzyme in endometrial cancer cell growth and differentiation [71–73]. The authors also showed that ERAP1 suppresses angiogenesis and endothelial cell migration in human endometrial carcinoma by regulating the angiotensin II concentration [72].

As stated before, variants of ERAP1 have been associated with decreased survival in cervical carcinoma, with ERAP1 loss being an independent predictor for survival, possibly due to the role of ERAP1 as a key determinant of the repertoire of MHC class I-presented peptides [53,74].

**Figure 2:**
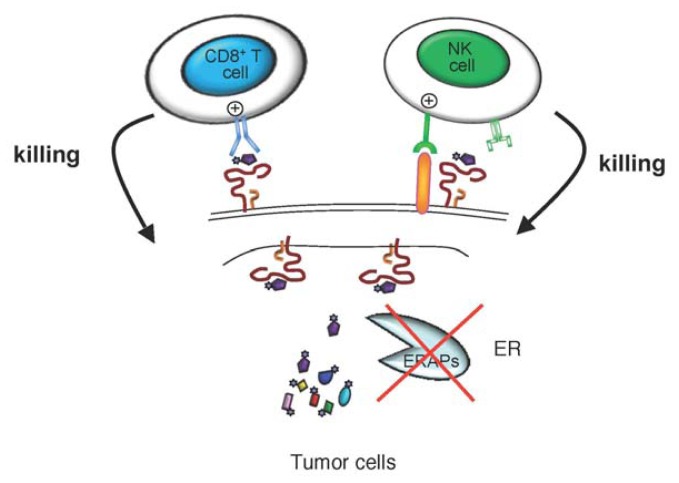
ERAPs inhibition: a possible novel strategy for anticancer immunotherapy. Antigenic peptides are generated in the cytosol and further trimmed at the NH_2_-terminus in the endoplasmic reticulum (ER) by ERAP1 and ERAP2 (ERAPs) aminopeptidases before being loaded onto MHC class I molecules. Peptide-MHC (pMHC) class I complexes are presented on the plasma membrane to be recognized by T cell antigen receptor on CD8^+^ T cells and by inhibitory receptors on NK cells. In the absence of ERAPs, a distinct repertoire of unstable pMHC class I complexes is produced and exported to the plasma membrane. These unstable complexes are sufficiently conformed to present antigens to CD8^+^ T cells but not enough to inhibit NK cells resulting in tumor-cell killing.

All these findings suggest that aberrant expression of ERAP1 may contribute to escape from immune surveillance. To evaluate the relevance of the ER peptide trimming inhibition on tumorigenicity, we stably reduced ERAP1 expression in a murine T-cell lymphoma by ERAP1-targeted small interfering RNA. We demonstrated that interfering with ERAP1 expression ultimately leads to tumor rejection in syngeneic animals by boosting NK cell-, and subsequently T cell-mediated cytolysis (Figure 2) [75]. This rejection was mainly due to NK cell response and depends on the MHC class I peptides presented by ERAP1-silenced tumor cells, because replacement of the endogenous peptides with high-affinity peptides was sufficient to restore an NK protective effect of MHC class I through the NK inhibitory receptor Ly49C/I. In spite of the relatively modest impact on overall MHC class I expression, we demonstrated that ERAP1 inhibition was able to shift the balance between activating and inhibitory signals towards NK cell activation resulting in target cell killing [75]. Thus, this was the first demonstration that tampering with ERAP1 activity via reduction of its expression can result in increased tumor immunogenicity *in vivo*, and may represent a novel pathway for anti-cancer therapeutic exploitation.

## 5. Conclusions

In this review, we have briefly summarized the recent knowledge on the biology of ERAP1 and ERAP2 enzymes and their possible links to human diseases.

In autoimmune diseases, association with HLA-class I risk alleles and their interaction with ERAP1 implicates aberrant ER peptide trimming leading to altered peptide presentation as the pathogenic mechanism. Additional functional studies are necessary in order to provide evidence on how ERAPs variants can affect disease predisposition and pathogenesis.

Loss of ERAP1 function has also been shown to substantially affect the presentation of epitopes by classical and nonclassical MHC class I molecules, probably contributing in some cases to the maintenance of chronic infection. In tumor cells, inhibition of ERAP1 expression has been shown to modify tumor immunogenicity by shifting the balance of activating and inhibitory signals towards NK cell activation resulting in target cell killing [75]. In contrast, the same mechanism was not shown in healthy cells lacking NK cell activating ligands such as splenocytes [34]. Based on these findings, it will be of interest to determine whether in humans, as previously shown in mice, manipulation of ERAPs could induce immune-mediated control of cancer. Thus, the possibility of targeting ERAPs through pharmacological or genetic modifications should be considered in order to provide novel anti-tumor immunotherapies.
